# Considerations for dairy cow nutrition in pasture-based systems

**DOI:** 10.3168/jdsc.2025-0992

**Published:** 2026-04-23

**Authors:** J.R. Roche, M. Dineen, J. Hills, J.K. Kay

**Affiliations:** 1 School of Biological Sciences, University of Auckland, Auckland, New Zealand 1010; 2 Teagasc, Animal and Grassland Research and Innovation Centre, Moorepark, Fermoy, Co. Cork, Ireland, P61 P302; 3 University of Tasmania, Cradle Coast Campus, Burnie, Tasmania 7320, Australia; 4 DairyNZ, Hamilton, New Zealand 3240

## Abstract

Pasture-based dairy systems offer a sustainable and cost-effective approach to milk production, particularly in temperate regions. This review synthesizes current knowledge on the nutritional profile of pastures and the nutritional management of grazing dairy cows, emphasizing the unique challenges and opportunities associated with using nonpasture feeds (i.e., grains, coproducts, or conserved forages: commonly referred to as supplements) to fill pasture deficits or increase milk production. Well-managed fresh pasture is a high-quality feed capable of supporting moderate milk production; it supplies an excellent profile of metabolizable AA and highly and rapidly digestible fiber. Its nutritional profile fluctuates, however, with regrowth stage and season, making precise diet management impractical. Our current understanding suggests that supplementary feeding (1) should first be employed to address deficits in ME during periods of low pasture growth; (2) at >30% of DMI can lead to nutrient imbalances, particularly in MP and AA; and (3) can reduce pasture utilization and farm profitability. There is no evidence to support individualized feeding strategies. Further research is required to understand the situations in which supplementary fat or crude protein and AA will increase milk production and to help better parameterize models for use with pasture-based systems.

## Highlights

• Well-managed pasture meets the requirements of moderate-yielding dairy cows.
• Metabolizable energy intake is the primary nutritional limit to milk production.
• Supplementation increases intake but reduces pasture intake and utilization via substitution.
• Protein and amino acids rarely limit milk unless supplements exceed ~30% of diet DM.
• Individualized concentrate feeding has not reliably improved herd milk production.

## Graphical Abstract

Pasture-based dairy systems aim to maximize pasture utilization while meeting cow nutrient requirements, with minimal supplementation. We evaluate the nutritional adequacy of well-managed temperate pasture and the role of supplementary feeding in grazing dairy cows. Fresh pasture is a high-quality, essentially complete feed for moderate-yielding cows, providing sufficient metabolizable energy (ME), metabolizable protein (MP), and highly digestible fiber. The primary nutritional limitation to milk production is ME intake, largely driven by constraints on dry matter (DM) intake during grazing. Supplementary feeds increase total intake and milk yield but reduce pasture intake through substitution, lowering pasture utilization and marginal milk production responses, particularly at higher pasture allowances. Diet protein, amino acids, or fat content rarely limit production unless supplements comprise more than ~30% of diet DM or pasture quality is unusually low. Despite variability in individual cow responses, individualized feeding strategies have not consistently improved herd-level performance in pasture-based systems.

**Figure:**
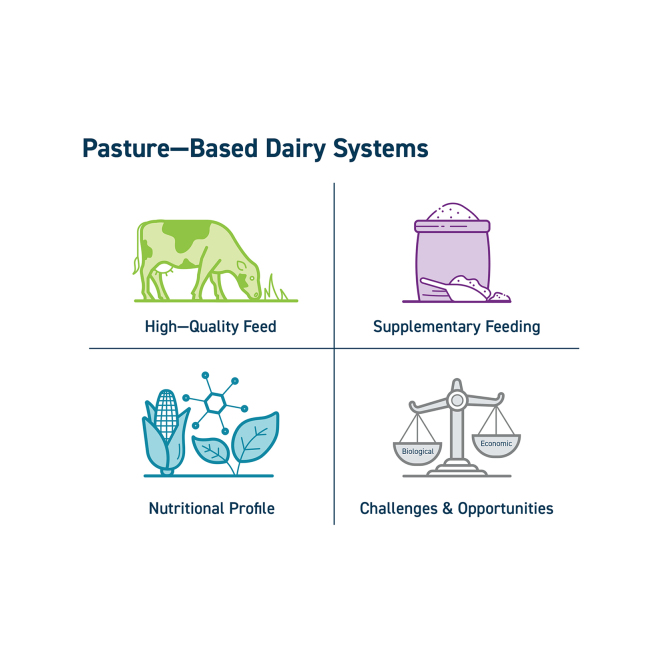

To maximize profitability, the dual biological aims of pasture-based systems are to (1) maximize the utilization of pasture grown per hectare, and (2) have the cow consume most of her diet from fresh pasture, with minimal use of labor, mechanization, and capital infrastructure for feeding (Dillon et al., 2005; Roche et al., 2017). To this end, pasture-based dairy systems are designed to synchronize the profile of pasture growth (i.e., feed supply) with herd feed requirement (i.e., feed demand; Roche et al., 2017; Spaans et al., 2019); this is achieved through seasonal and compact-calving (herd calving in ≤12 wk) in late winter and early spring.

Despite similarly shaped profiles for pasture growth in temperate environments and cow DMI, there are periods when pasture growth is not adequate to meet herd DMI requirements and conserved forages, grains, or coproducts (i.e., supplements) are offered in addition to available pasture (Roche et al., 2017). A further reason for providing supplements is sometimes postulated: to “complement” the pasture, suggesting that temperate pastures are not a complete feed (Roche, 2017). Considering the low NFC, high NDF, high soluble CP, and low to moderate fatty acid (**FA**) concentrations (Roche et al., 2009) relative to TMR, this postulate is worth considering in any review of the nutrition of pasture-based cows. Some farmers include a low rate of supplement in the diet of cows as an easy way to supplement cows with minerals, particularly in early lactation. In this review, we will focus on the feed quality of fresh pasture for moderate yielding dairy cows (i.e., <25–30 kg FCM at peak) and the effects of providing supplements on pasture and total DMI, pasture substitution, and pasture utilization, and ultimately, the effect on milk production.

There is a strong relationship between the proportion of the diet that is grazed pasture and the cost of producing a kilogram of milk (Dillon et al., 2005) and between the cost of producing milk and farm profit per hectare (Neal and Roche, 2019). Pasture utilization per hectare has the strongest biophysical relationship with profitability in reported analyses (Ramsbottom et al., 2015; Neal and Roche, 2019) and utilization is affected by pasture management and the use of supplements. Production efficiency and profitability are dependent on ensuring the correct supplement is used at the correct time.

On the correct supplement, as with any biological system, factors limiting milk production align with Von Liebig's principle of the minimum (Von Liebig, 1840): production is constrained by the first most limiting factor. To define the “cascade of nutritional limitations” (Roche, 2017), it is important to understand the nutritional composition of fresh pasture relative to the nutrient demands of the dairy cow at different stages of lactation and pregnancy. From a nutritional perspective, ME and MP requirements for maintenance, milk production, reproduction, BW or BCS gain, and per kilogram of metabolic BW (kg^0.75^) are comparable for cows grazing fresh pasture with those established for housed dairy cattle fed a formulated TMR, but with additional ME required for grazing and walking (Roche, 2017). We will not consider these requirements further.

Roche (2017) made the case for fresh temperate pasture being a complete feed, providing sufficient ME, CP, and macro- and micronutrients for moderate peak milk production (i.e., 18 kg of DMI; 25–30 kg of FCM; 2.0 to 2.5 kg of milk fat and protein). Organic matter total-tract digestibility (**TTD**) of temperate pasture species is >80% (Morgan and Stakelum, 1987; Rius et al., 2012; Garry, 2016) and in vivo flow studies have demonstrated that more than 70% of pasture NDF is digested in the rumen, and with over 80% TTD (Dineen et al., 2020). Recent in vitro research corroborates these findings (Dineen et al., 2021b; Heffernan et al., 2025a; Figure 1) as does the meta-analysis by Huhtanen et al. (2010). In summary, there is compelling evidence that temperate pastures undergo both rapid and extensive NDF digestion, characteristics that promote high flows of microbial protein, enhancing the supply of both ME and MP (O'Mara et al., 1997; Roche, 2017; Dineen et al., 2021a), assuming no deficiency in CP and RDP.

**Figure 1:**
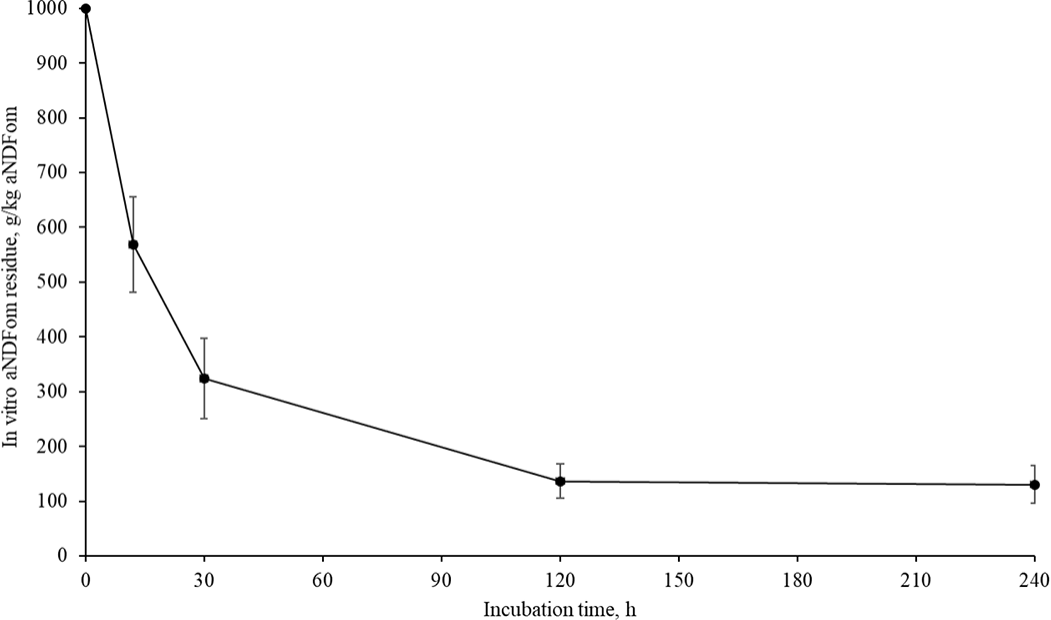
In vitro amylase- and sodium sulfite-treated NDF corrected for ash residue (aNDFom) after fermentation for 12, 30, 120, and 240 h. Data are mean ± SD (error bars) for 198 pasture samples collected on 12 Irish dairy farms over a 2-yr period as part of Heffernan et al. (2025a). Pasture samples were analyzed for digestibility of aNDFom in accordance with Raffrenato et al. (2018), with the inclusion of a 12-h time point as described by Dineen et al. (2021a).

Achieving high pasture utilization, cow DMI, and high OM TTD requires careful pasture management. There is a positive relationship between daily pasture allowance and DMI, but a negative relationship with pasture utilization (Wales et al., 1999). There is also a positive relationship between utilization and subsequent OM TTD (Stakelum and Dillon, 1990). Pasture management is, therefore, a compromise between achieving high DMI per cow in the current grazing event and maintaining high pasture utilization and feed quality for subsequent events. Lee (2009) concluded that optimum pasture and animal production occurred when (1) perennial temperate pastures were grazed between emergence of the second and third leaf (see Fulkerson and Donaghy, 2001); and (2) an allowance that resulted in postgrazing residual heights between 35 and 40 mm.

Consistent with the premise that well-managed temperate pastures are a complete feed, Kolver and Muller (1998) reported that ~88% of the difference in milk yield between TMR-fed cows and those grazing a pasture could be explained without any attribution to nutritional deficiencies in the pasture; their modeling indicated that only ~12% of the difference in milk production was attributable to differences in the nutrient profile, most specifically excess CP, but potentially NDF to NFC ratio and its effect on N digestion and metabolism. Their results suggest that the “complementarity” postulation previously mentioned will result in limited gains in milk production per cow; instead, any real gains from supplementary feeding will come from increasing DMI and ME intake. It is worth considering, however, that cows are heterogeneous in their response to supplements (Fulkerson et al., 2008). The marginal milk production response (**MMPR**) to supplements (i.e., kg FCM/kg supplement DM) may, therefore, be greater if individualized feeding strategies are used to best effect. Use of supplements to increase DMI or provide specific limiting nutritional factors as well as individualized feeding strategies will be further considered.

Two facts are unequivocal: (1) grazing cows consume less than TMR-fed cows (Kolver and Muller, 1998); and (2) when cows are offered supplements, in addition to their grazing allocation, total DMI is increased (Bargo et al., 2003), with few exceptions. The regulation of DMI in mammals is complex, and although the neuroendocrine control mechanisms are conserved across mammalian systems (Roche et al., 2008), the requirement for the cow to work to consume her feed, the perishable nature of the fresh pasture, if not consumed, and a consequential reduction in subsequent feed quality through fiber proliferation if pasture is not consumed in the grazing event (i.e., before moving to the next pasture) complicate matters further in pasture-based systems. Efforts to increase DMI in grazing cows cannot result in underutilized pastures.

The reason for the lower DMI in grazing cows is not immediately clear. One factor is the act of grazing, with cows eating more fresh pasture when housed and offered cut pasture than when grazing the same pasture (Boudon et al., 2009). This may be due to the greater time needed to graze, a process that requires active selection and prehension, compared with consuming cut pasture from a trough; it may be tied to circadian rhythms, with photoperiod known to affect DMI and milk production (Dahl et al., 2000); or it may be a result of nutritional characteristics of grazed pastures that limit DMI or, more importantly, the intake of ME, and could be overcome through the provision of supplements.

Neural and endocrine networks integrate environmental and metabolic and physical information and signal the arcuate nucleus of the hypothalamus to stimulate hunger- or satiety-related feeding behaviors (Roche et al., 2008). In general, hunger and satiety are a balance between cues stimulated by the amount and nutritional composition of what has been consumed, but these are modified, at least in part, by environmental signals. Cows are diurnal animals, with tendencies toward crepuscular behaviors (Hafez, 1969; Sheahan et al., 2011): they consume most of their feed during daylight, but with particularly large periods of grazing activity at dawn and dusk. Behavioral analyses identify these trends clearly (Hafez, 1969; Sheahan et al., 2011) and they are evident in early, mid, and late lactation (Sheahan et al., 2011), with sunrise and sunset natural signals to start and cease eating. Shorter days in spring during peak milk production may, therefore, limit pasture DMI. In housed dairy systems, artificial lighting is used to manipulate this diurnal effect on DMI (Dahl et al., 2000), with longer day scenarios increasing milk production and the increase in milk production resulting in an increase in DMI. This artificial manipulation of photoperiod in housed situations could be one reason for the difference in DMI between grazing cows and those being fed TMR (Kolver and Muller, 1998).

Another reason hypothesized for the lower DMI of pasture relative to TMR is the higher NDF in pasture. Given the rapid and extensive NDF digestion of temperate pastures under well-managed conditions, however, physical fill is unlikely to constrain DMI. Dineen et al. (2020) concluded similarly from an assessment of rumen pool size measurements. Further, Vazquez and Smith (2000) reported that pasture NDF explains less than 10% of the variation in pasture DMI. Fiber, therefore, is unlikely to be a nutritional variable limiting DMI in temperate pasture-based systems.

As day length limits pasture DMI, it could be hypothesized that the provision of supplements that take very little time to consume (e.g., concentrate feed in the parlor) could lead to a significant increase in DMI, without reducing pasture utilization. Scientific reports on the effects of supplementing grazing cows on total DMI and milk production concur (Stockdale, 2000; Bargo et al., 2003; Macdonald et al., 2017; Roche, 2017), but with important caveats. Cows consuming supplement reduce their time spent grazing and, as a result, their pasture DMI; this phenomenon is referred to as substitution and the reduction in pasture DMI (pasture refused) divided by the supplement DMI is known as the substitution rate (**SR**; Table 1; Stockdale, 2000). Despite this, total DMI and milk production are generally greater than in unsupplemented cows (i.e., SR is rarely >1), but the DMI and MMPR are not as great as expected from the additional DM offered.

**Table 1:** Estimated substitution rate and marginal milk production response (MMPR) to supplementary feeds (kg/kg DM) for 500-kg dairy cows at BCS 4 and offered 1, 3, 5, or 7 kg of DM concentrate while consuming 5, 10, 15, or 20 kg of DM pasture^1^, ^2^

Supplementary feed DMI, kg/d	Season	Pasture DMI, kg/d							
		Substitution rate at pasture DMI			MMPR at pasture DMI				
		5	10	15	20	5	10	15	20
1	Spring	0.12	0.28	0.44	0.60	0.88	0.76	0.63	0.50
3	Spring	0.18	0.34	0.50	0.66	0.84	0.71	0.58	0.45
5	Spring	0.24	0.40	0.56	0.72	0.79	0.66	0.53	0.40
7	Spring	0.30	0.46	0.62	0.78	0.74	0.61	0.48	0.36
1	Summer	0.01	0.17	0.33	0.49	1.25	1.12	1.00	0.87
3	Summer	0.07	0.23	0.39	0.55	1.20	1.08	0.95	0.82
5	Summer	0.13	0.29	0.45	0.61	1.16	1.03	0.90	0.77
7	Summer	0.19	0.35	0.51	0.67	1.11	0.98	0.85	0.72
1	Autumn	—	0.06	0.22	0.38	1.34	1.21	1.08	0.96
3	Autumn	—	0.12	0.28	0.44	1.29	1.16	1.04	0.91
5	Autumn	0.02	0.18	0.34	0.50	1.24	1.12	0.99	0.86
7	Autumn	0.08	0.24	0.40	0.56	1.20	1.07	0.94	0.81

1 Values less than zero from the equations are omitted as outside the range of values tested.

2 Predicted from equations reported in Stockdale (2000), using a 500-kg cow BW, reported pasture and supplementary feed DMI, and calendar season.

Substitution rate and the MMPR to supplements are dependent on situational factors (Table 1; Stockdale, 2000): (1) pasture and concentrate DMI: SR increases and MMPR declines with pasture and concentrate DMI; (2) the BW of the cow (as a proxy for genetic merit): the higher the BW, the lower the expected SR and the greater the expected MMPR to supplements (Fulkerson et al., 2008); (3) season of year: SR is greater and MMPR less in spring than summer, and in summer than autumn; and (4) the percent legume in the pasture: SR is greater and MMPR less in grass-dominant pastures than legume-dominant; grass type (temperate vs. tropical) does not influence SR, although when the base forage is a tropical species, the ME and TTD of OM and NDF is less and the MMPR to supplements would be expected to be ~20% greater than reported on temperate pastures. Although supplement type (forage vs. concentrate; starch-based vs. fermentable fiber-based) has been reported to affect SR and MMPR (see Bargo et al., 2003), the reported effects are inconsistent and were not included in the most complete multivariate equation (Stockdale, 2000).

The reasons for the effects of supplements on pasture DMI are not completely understood, but circulating concentrations of ghrelin decline when cows consume supplements during milking (Roche et al., 2007). This indicates a lowering of the endocrine basis for hunger with prior nutrient consumption (Roche et al., 2008) and coincides with the reduced grazing activity postsupplementation reported by Sheahan et al. (2011). Irrespective of the reasons, the literature is consistent in that SR is least and MMPR to supplements greatest in autumn and at low pasture allowances, and that SR is greatest and the MMPR least in spring, all other things being equal. Further, SR increases and MMPR declines with pasture and supplement DMI (Stockdale, 2000; Bargo et al., 2003; Kay et al., 2022).

Although DMI explains two-thirds of the milk production differences between pasture-grazed and TMR-fed cows (Kolver and Muller, 1998), differences in nutritional composition are also worth exploring. It could be hypothesized, for example, that low levels of NFC and lipids or high levels of CP are contributing to milk yield differences. A stark contrast between grazed pasture and TMR is the low levels of NFC in pasture DM. Greater NFC content would be expected to increase the stochiometric efficiency of rumen fermentation, reduce acetate to propionate ratio and the losses associated with methane production, and increase milk volume and protein yield (Roche et al., 2010). Increasing diet NFC content in an energy-maintained diet, however, has not confirmed this. In some studies, perennial ryegrass cultivars selected for increased NFC concentrations increased DMI and milk production (Miller et al., 2001; Moorby et al., 2006), but the low pasture CP (9.2%–10.6% DM) made it difficult to extrapolate to realistic scenarios. Subsequent experiments conducted under more representative pasture CP concentrations found no effect of pasture NFC on DMI or milk production (Taweel et al., 2005; 2006; Tas et al., 2006). Supporting this, Carruthers et al. (1996), Roche et al. (2010), and Higgs et al. (2013) identified no differences in yield of milk components and only minor changes in milk yield and milk composition when the carbohydrate profile of grazing diets shifted from fermentable fiber-based feeds to NFC-based feeds, so long as ME intake remained the same. Similarly, in a multiyear farm systems experiment involving an increase in diet NFC through supplementation, Macdonald et al. (2017) reported the same production of milk fat and protein with grain- and silage-based supplements when differences in the ME content of the supplement were considered: cows produced 7.6 g of milk fat and protein for every extra megajoule of ME consumed. When considering the “cascade of nutritional limitations” (Roche, 2017), the supply of dietary ME is, therefore, more important than the source and composition of the feed component supplying the ME.

If ME is the first limiting factor (Kolver, 2003; Roche, 2017), fat is an energy-dense component that, when rumen inert, can increase milk production. In TMR diets, fat can be used to increase ME when diet NFC and NDF are already nearing limits for sustaining rumen health and function. Roche et al. (2009) reported that ether extract concentration in pasture ranged from 4.0% to 4.4% DM throughout the year. They also reported that although the relationship between pasture ME and ether extract concentration was positive and statistically significant, pasture ether extract explained <3% of the variation in pasture ME. This small coefficient of determination probably reflects the relative lack of variation in pasture ether extract, rather than a lack of effect of fat content on pasture ME. Recent plant breeding efforts have focused on increasing the fat content of pasture as a strategy to enhance its energy density (Winichayakul et al., 2020) and as a prospective methane mitigant (Grainger and Beauchemin, 2011).

The benefits from increased fat consumption in pasture-based systems, however, are unclear. A limited number of studies have investigated the effects of supplementing fat to pasture-based cows. Kay et al. (2006) provided early-lactation cows with 540 g/d of a palmitic-oleic acid mix for the first 36 DIM. Mean milk yield increased from 19.4 to 22.5 kg/d (+3.1 kg), but fat and protein percentage declined with the fat supplement, and milk component yield was not significantly affected. In comparison, Heffernan et al. (2025b) investigated the supplementation of 200 to 240 g of palmitic acid or a palmitic-oleic acid mix in established lactation (from ~65 DIM to ~180 DIM) and reported a 0.8 kg increase in ECM in the treatment group receiving the mix of FA, but no ECM increase in the cows receiving the palmitic acid supplement. The increase in ECM was due to a 40 g increase in milk fat yield associated with fat supplementation. Although not significant in their study, Kay et al. (2006) also reported a 47 g increase in milk fat yield in their cows consuming the palmitic-oleic acid mix. The results suggest that milk fat production may increase with greater fat intake, but the effect appears dependent on the source of fat and stage of lactation. Further research should focus on different fats at different stages of lactation to understand the effects of type of fat and the level and timing of supplementation on the MMPR to fat supplements.

The CP concentration of temperate pastures has traditionally been considered sufficient to sustain milk production in grazing dairy cows (Bargo et al., 2003; Roche, 2017). However, low-CP pastures (<16% of DM) can occur during late spring and summer on some farms (Heffernan et al., 2025a). Furthermore, extensive ruminal proteolysis and preduodenal losses of consumed protein have been widely reported (Dineen et al., 2020, 2021a; Morales et al., 2024). Beever and Siddons (1986) estimated that only 3% to 5% of MP originates from undegraded pasture protein and Dineen et al. (2021a) demonstrated that cows consuming fresh pasture exhibit a large dependence on microbial AA to support MP supply. These results suggest that MP supply could limit animal performance at lower pasture CP concentrations (<20% DM in early lactation) or when cows consume low N supplements (Macdonald et al., 1998). Studies have demonstrated milk yield responses when grazing cows were supplemented with undegraded dietary protein in the form of rumen-protected casein (Rogers et al., 1980; Minson, 1981), formaldehyde-treated soybean meal (Delaby et al., 1995; Delagarde et al., 1997), or fishmeal (Macdonald et al., 1998; O'Mara et al., 2000). We, therefore, consider MP as the next variable in the “cascade of nutritional limitations.”

Kolver (2003) simulated changes to DMI and feed composition using the Cornell Net Carbohydrate and Protein System (CNCPS) model. Although acknowledging that ME was the first limiting factor for milk production, MP-allowable milk production was greater than AA-allowable milk production in pasture-fed cows producing >40 kg of FCM. This is much greater production than would be expected in pasture-fed cows and field studies confirm the lack of effect of AA under normal grazing conditions; Younge et al. (2000) reported that rumen-protected Met+Lys did not increase milk yield or composition in mid-lactation cows grazing high-quality grass, suggesting AA were not the current constraint under those conditions. The points at which milk production, pasture CP, and diet composition result in AA limiting milk production requires further evaluation to enable better parameterization of nutrition simulation models for use in pasture-based systems.

In summary, although temperate pastures are acknowledged to be of high nutritional quality, with extensive and rapid digestion of NDF, and they provide a reasonable supply of MP to the small intestine, it is acknowledged that (1) pasture DMI limits milk production, and (2) seasonal variation in pasture DM growth mean there is a need to supplement dairy cows at key times of lactation in most systems. The “cascade of nutritional limitations” indicates that the key nutritional factor limiting production is ME and, except in situations where supplements exceed ~30% of diet DM, CP and AA are rarely limited. However, the MMPR to supplements is dependent on the relative feed deficit experienced, with responses declining at greater pasture DMI.

There may be an opportunity to use the supplements employed to greater effect. For example, identifying cows that have greater MMPR to supplements and offering these cows a greater proportion of the supplements has been postulated for decades to be a strategy for improving herd responses to supplements (Leaver, 1988; Hills et al., 2015). Hills et al. (2015) presented a comprehensive review of the literature on the effects of individualized feeding on individual cow- and herd-level MMPR to supplements. Nevertheless, we believe a short summary of individualized feeding strategies is appropriate.

In pasture-based dairy systems, concentrate supplements, comprising mixtures of grains and coproducts, are often fed during milking to increase DMI and, consequently, milk production (Bargo et al., 2003). Before the computerization of in-parlor feeding systems and sensor and communication technologies, these were offered so that all cows received the same amount of supplement (i.e., flat-rate feeding strategy; **FR**). But, with the rapid increase in cow identification technology, coupled with measurement, sensor, and communication technologies, and more recently the advent of analytical and generative artificial intelligence and machine learning, there is increased interest in the potential benefits of individualized feeding strategies, such as feeding each cow in the herd relative to some individual characteristic (e.g., milk yield; feed-to-yield strategies, **FtY**). The basis of the FtY hypothesis is that variation between cows in their MMPR to supplements can be defined by some measurable characteristic, and the optimization of feed supplement allocation at an individual cow level will increase the herd's total MMPR to supplements (Dela Rue and Eastwood, 2017).

In support of the hypothesis that individualized allocation of supplements will increase herd MMPR, cows with a greater EBV for milk production have (1) greater circulating ghrelin concentrations before and after meals (Roche et al., 2006, 2007), indicating a greater physiological hunger before and after meals; (2) present with lower SR (Linnane et al., 2004; Sheahan et al., 2011); and (3) have greater MMPR to supplements (Fulkerson et al., 2008; Macdonald et al., 2008). Raedts and Hills (2024) compared 2 concentrate levels (2 vs. 6 kg DM/d) over an 8-mo period in cohorts of cows differing in breed/genotype, BW, and early-lactation milk yield and reported significant variation in MMPR among cohorts (ranging from 0.18 to 1.22 kg/kg DM extra concentrate). The highest MMPR were in Holstein-Friesian cows compared with cross-bred cows, and in cows with low initial milk yield or BW. Raedts and Hills (2024) also reported reduced grazing time at the higher concentrate allowance, but the greatest reduction in grazing time was evident in the cross-bred cows, reinforcing the premise that the lowest MMPR was due to greater SR. Collectively, the results support the potential for individualized and targeted feeding of supplements, such that if cows with greater MMPR receive greater amounts of supplement, herd total MMPR should be increased.

Despite the heterogeneity of MMPR to supplements within herd and the potential to exploit this to increase herd MMPR, the overwhelming conclusion from studies comparing FR with FtY is that individualized allocation of supplements according to milk yield does not increase herd MMPR (Leaver, 1988; Hills et al., 2015). It was hypothesized that the reason for the lack of effect might be due to experimental design, where unlimited pasture availability in most studies mitigated any potential effect of FtY. Support for this hypothesis came from García et al. (2007), wherein cows on a restricted allowance of lucerne pasture and fed a concentrate supplement based upon individual cow milk yield produced 7% more fat and protein compared with FR cows.

In practice, pasture allowance is restricted to ~90% of herd voluntary DMI to ensure pasture utilization is high and long-term feed quality is maintained. Dry matter intake as a percentage of voluntary DMI is negatively associated with the “relative feed deficit” experienced by the cow (Poole et al., 2019). A restricted pasture allowance creates competition between cows for a limited resource and leads to variation in the relative deficit for individual cows within a herd, possibly affecting their MMPR. For example, Poole et al. (2019) reported that MMPR increased 9% for every 10 mm decrease in postgrazing residual (a proxy for relative feed deficit). It is plausible that cows with high EBV for milk production experience greater relative feed deficit compared with lower EBV herd-mates, and individual and, therefore, herd MMPR to supplements would be greater if they were to receive a greater proportion.

Recent studies have examined individualized feeding strategies under restricted grazing conditions (Brady et al., 2022; Dela Rue et al., 2025). Brady et al. (2022) investigated the interaction between concentrate feeding strategies and dairy cow genotypes under restricted grazing (pasture allowance of 13 kg DM/d at peak lactation) and drought-stressed conditions. Although the authors reported the FtY herd produced more milk than the low FR treatment group, the experimental design was confounded because the FtY treatment also received more concentrate DM than the FR herd. It is, therefore, not possible to separate the effect of feed amount from feeding strategy. More recently, Dela Rue et al. (2025) reported on whether individualized feeding of concentrate supplements based on milk yield could enhance milk production in pasture-based cows near peak lactation, but with the same total amount of concentrate made available to both treatments. This study, conducted under typical restrictive conditions of practical rotational grazing, identified no significant herd-level benefit from FtY compared with FR when the total concentrate offered was ≤6 kg DM/cow per day and average milk yield was ≤30 kg/d. To date, there is little evidence that FtY strategies based on cow milk yield provide any increase in herd MMPR compared with FR strategies, irrespective of pasture allowance. But, strategically targeting individuals or cohorts of cows may still have benefits and further research on the effects of genotype and milk yield, stage of lactation, pasture allocation and grazing conditions, including due to weather, and under different pasture quality and supplementary feeding scenarios is warranted.

In summary, pasture-based systems have 3 unique features that must be considered from a nutrition perspective. First, pasture is a “fresh” feed and is, therefore, perishable; if it is not used, it decays in the paddock and is wasted (Roche, 2017). In addition, its decay results in lower pasture quality in subsequent grazing events (Stakelum and Dillon, 1990). Second, the first limiting nutritional variable in the “cascade of nutritional limitations” is ME and other nutritional factors are unlikely to limit production until supplements are >30% DMI. Third, when a cow consumes supplements, her physiological hunger signals are abated (Roche et al., 2007), and she reduces her grazing time (Bargo et al., 2003; Sheahan et al., 2011, 2013) and her DMI of pasture (Stockdale, 2000). This directly affects the MMPR to supplements. Individualized feeding strategies have not, as yet, been reported to increase MMPR.
